# Cautionary notes on the use of NF-κB p65 and p50 antibodies for CNS studies

**DOI:** 10.1186/1742-2094-8-141

**Published:** 2011-10-14

**Authors:** Miles Herkenham, Priyanka Rathore, Pierre Brown, Samuel J Listwak

**Affiliations:** 1 Section on Functional Neuroanatomy, Laboratory of Cellular & Molecular Regulation, National Institute of Mental Health, NIH, Bethesda, MD 20892, USA

**Keywords:** NF-κB, transcription factor, immunohistochemistry, antibody specificity

## Abstract

**Background:**

The characterization and cellular localization of transcription factors like NF-κB requires the use of antibodies for western blots and immunohistochemistry. However, if target protein levels are low and the antibodies not well characterized, false positive data can result. In studies of NF-κB activity in the CNS, antibodies detecting NF-κB proteins have been used to support the finding that NF-κB is constitutively active in neurons, and activity levels are further increased by neurotoxic treatments, glutamate stimulation, or elevated synaptic activity. The specificity of the antibodies used was analyzed in this study.

**Methods:**

Selectivity and nonselectivity of commonly used commercial and non-commercial p50 and p65 antibodies were demonstrated in western blot assays conducted in tissues from mutant gene knockout mice lacking the target proteins.

**Results:**

A few antibodies for p50 and p65 each mark a single band at the appropriate molecular weight in gels containing proteins from wildtype tissue, and this band is absent in proteins from knockout tissues. Several antibodies mark proteins that are present in knockout tissues, indicating that they are nonspecific. These include antibodies raised against the peptide sequence containing the nuclear localization signals of p65 (MAB3026; Chemicon) and p50 (sc-114; Santa Cruz). Some antibodies that recognize target proteins at the correct molecular weight still fail in western blot analysis because they also mark additional proteins and inconsistently so. We show that the criterion for validation by use of blocking peptides can still fail the test of specificity, as demonstrated for several antibodies raised against p65 phosphorylated at serine 276. Finally, even antibodies that show specificity in western blots produce nonspecific neuronal staining by immunohistochemistry.

**Conclusions:**

We note that many of the findings in the literature about neuronal NF-κB are based on data garnered with antibodies that are not selective for the NF-κB subunit proteins p65 and p50. The data urge caution in interpreting studies of neuronal NF-κB activity in the brain.

## Background

NF-κB is a transcription factor that is ubiquitously present in all cells of the body. It exists as a homo- or hetero-dimer comprising typically p50 and p65 (RelA) subunits, but also combinations of these subunits with other members of the Rel family, such as p52, c-Rel and RelB [1]. Activation of NF-κB by enzymatic degradation of the bound inhibitory protein, predominantly IκBα, results in exposure of the nuclear localization signal (NLS) on p50 and p65, allowing movement of the subunits from the cytoplasm to the nucleus where they bind to consensus κB sequences in the DNA. Characterization of this activity is afforded by the use of antibodies that recognize and mark the proteins in western blots of cytoplasmic and nuclear protein fractions. Antibodies are used also in EMSA supershift and immunoprecipitation experiments, both of which are commonly used to study transcription factor activity. Identification of the cell types showing activity is achieved by microscopic localization of the antibody-tagged subunits with immunohistochemistry (IHC) or immunocytochemistry (IC). In the NF-κB/Rel field, numerous commercial and non-commercial antibodies have been raised against all the subunits and also against activated (e.g., phosphorylated) forms of the molecules.

NF-κB function is most studied in the immune system [2], but it has been shown to be present in the brain, in both neurons and non-neuronal cells, notably glia [3]. Of the main techniques for measuring NF-κB activity, most lack the ability to distinguish the cell types activated. Microscopic techniques that can distinguish cell types include in situ hybridization histochemistry (ISHH), which localizes changes in gene transcription levels in cells, and IHC/IC, which identifies protein locations and levels in phenotyped cells. After NF-κB was identified as a CNS transcription factor, studies on its localization in the nervous system blossomed. Many of the studies painted a complex and contradictory picture of NF-κB function in the CNS. Strikingly, whereas ISHH of IκBα mRNA transcription indicated that NF-κB activity was confined to non-neuronal cells, IHC painted a different picture, showing neuronal as well as non-neuronal staining of NF-κB subunits in various paradigms and assays.

All of the techniques that rely on antibodies require antibody specificity to ensure that the assay is truly tracking NF-κB proteins. An antibody is specific if it recognizes and binds to the epitope in the target protein and to no other molecular or nonspecific entities. Validation of antibody specificity for IHC is typically done by a set of control experiments that involve omission of the primary antibody and co-incubation of the preparation with an excess amount of the peptide used for immunization. Another important test of specificity is the demonstration in western blot that the antibody binds to a single protein that runs in the gel at a molecular weight that is expected of the target molecule. The most stringent of control tests is the demonstration that the binding or staining of the antibody is absent in tissues or cells from a genetic mutant animal that has been engineered to lack that protein, i.e., a knockout (KO) mouse. Surprisingly, there are ample cases of antibodies that "pass" all the control tests except the last one. Thus, this study used the resource of tissue from p50 KO and p65 KO mice to test a number of widely used NF-κB antibodies. Only a few antibodies passed all of the tests, and several widely used antibodies failed. The most significant failures were antibodies raised against the region containing the NLS of both p65 and p50. A review of the literature on NF-κB in brain reveals that most of the claims of constitutive or induced NF-κB activity in neurons are based on results obtained with antibodies that failed the rigorous specificity test.

## Methods

### Animals

C57BL/6 mice were obtained from The Jackson Laboratories. The *rela*^-/-^/*tnfr1*^-/- ^(p65 KO) mice were a gift of Mollie Meffert [4,5], and *nfkb1*^-/- ^(p50 KO) mice were a gift of Christopher Hunter [6,7]. In both mice, the gene deletion results in complete elimination of the entire protein. The p50 KO mouse also lacks p105, the longer bioactive *nfkb1 *gene product and precursor to p50, but for clarity in the text, only the p50 KO is noted in the designation. The p65 KO mouse has additional deletion of the Type 1 TNF receptor, which permits survival of the mouse into adolescence [8], and for clarity, the p65/TNFR1 double KO is called the p65 KO. All animal procedures were approved by the Animal Care and Use Committee of the Intramural Research Program, NIMH.

### Antibodies used

Antibodies directed against p65 and p50 were obtained from commercial sources (Santa Cruz Biotechnology, Santa Cruz, CA; Chemicon and Upstate (Millipore), Billerica, MA; Calbiochem, St. Louis MO; Cell Signaling, Beverly, MA; Abcam, Cambridge, MA; Epitomics, Burlingame, CA) or from the NIH, including a p65 antibody that was a gift of Ulrich Siebenlist, National Institute of Allergy and Infectious Diseases, NIH, and antibodies deposited by Dr. Nancy Rice, National Cancer Institute, NIH, into the repository of the Biological Resources Branch, NCI, Frederick, MD (http://web.ncifcrf.gov/research/brb/reagents/AntiseraReagent.aspx). The list of antibodies tested for the study is provided in Table 1.

**Table 1:** NF-κB Antibodies tested for specificity in p65 KO and p50 KO tissues

**Catalog Name**	**Target**	**Source**
**Anti p65 antibodies (C-terminus-directed listed first)**		
sc-372G/R	Goat or rabbit polyclonal to aa 531-550 (C-20)	Santa Cruz
NIH p65 (7057)	Rabbit polyclonal to aa 538-550	NIH-Siebenlist
PC137	Rabbit polyclonal to C-term (within the last 60 aas) Same as Chemicon (AB 1604), Rockland (100-4165), and Upstate (now Millipore)	Calbiochem
1546	Rabbit monoclonal to C-terminus	Epitomics
2A12A7	Mouse monoclonal to aa 375-550	Invitrogen
3034	Rabbit polyclonal to region around Ser276	Cell Signaling
4764	Rabbit monoclonal to N-terminus	Cell Signaling
sc-109	Rabbit polyclonal to N-terminus	Santa Cruz
sc-8008	Monoclonal to N-terminal aa 1-286	Santa Cruz
		
**"Activated" p65 antibodies**		
MAB3026	Clone 12H11; monoclonal to region of NLS (formerly Boehringer and Roche #1697838; originally Kaltschmidts' a-p65M)	Chemicon
3037	Rabbit polyclonal to phospho-Ser276-p65	Cell Signaling
2615	Rabbit polyclonal to phospho-Ser276-p65	Abcam
		
**Nancy Rice (NCI, Frederick, MD repository) rabbit polyclonal p65 antibodies**		
1226-TB3	C terminus	NCI repository
1774 TB11	downstream of NLS	NCI repository
1207-TB4	N terminus	NCI repository
33-TB4	downstream of NLS (mouse sequence)	NCI repository
		
**Anti p50 antibodies**		
ab7971	Rabbit polyclonal to aa 330-433	Abcam
sc-8414	Monoclonal to aa 120-239	Santa Cruz
06-886	Rabbit polyclonal to aa 1-12	Upstate
3035	Rabbit polyclonal to N-terminal	Cell Signaling
1559	Rabbit monoclonal between aa 340-370	Epitomics
		
**"Activated" p50 antibody**		
sc-114	Rabbit polyclonal to region of NLS (at 357-365)	Santa Cruz
		
**Nancy Rice (NCI, Frederick, MD repository) rabbit polyclonal p50 antibodies**		
1263-TB7	N terminus of mouse p50	NCI repository
1157-TB5	upstream of NLS	NCI repository
1613-TB7	NLS	NCI repository

Manufacturers' information about location of amino acid (aa) sequences of the human protein that the antibody was raised against.

### Primary cell culture

#### Neurons

Mouse neurons were cultured from 16-day embryonic C57BL/6 mouse brains as described previously [9]. Hippocampi or cortices were dissected out in cold Hanks balanced salt solution (HBSS) after careful removal of meninges and were trypsinized (final 0.05% concentration) for 15 min at 37°C. The digested tissues were washed twice with Dulbecco's modified Eagle's medium (DMEM) (Invitrogen, Carlsbad, CA) containing 10% fetal bovine serum (FBS) (Invitrogen) and triturated. Large cellular debris was removed using a 40-μm nylon strainer, and smaller cellular debris was removed by centrifuging at 200 × g for 5 minutes. The pellet was resuspended in Neurobasal medium supplemented with B27 (1X), Glutamax (2 mM), penicillin (100 U/ml), and streptomycin (100 mg/ml) (all from Invitrogen) and seeded onto poly d-lysine coated coverslips at a density of 0.10 × 10^6 ^cells/well for immunocytochemistry and in 6-well plates at 2 × 10^6 ^cells/well for western blots. Neurons were maintained at 37°C in the presence of 5% CO2/95% O2. After four days in culture, the medium was changed to fresh supplemented Neurobasal medium in some experiments containing also cytosine arabinoside (araC) (10 mM) and 2-deoxycytidine (100 mM) (Sigma) to inhibit astrocyte growth. After 10 days in culture, the cells were subjected to the various experimental conditions and processed for either immunostaining or immunoblotting.

#### Mixed brain cells

Mouse brain cells comprising astrocytes, microglia, fibroblasts, and some neurons were cultured from 16-day embryos. Subcortical pieces were dissected out in cold HBSS, homogenized, pelleted, resuspended, and plated for 10 days as above except the medium contained only DMEM with 10% FBS, penicillin (100 U/ml), and streptomycin (100 mg/ml).

### Kainic acid (KA) treatment of animals

Mice were maintained in the animal colony under standard housing and ad lib feeding conditions. Kainic acid (Tocris, Ellisville, MO) was dissolved in sterile water immediately before use. From a stock solution of 0.3 mg/ml, 1.0 μl was slowly infused with a Hamilton syringe stereotaxically lowered to the coordinates A-P +1.5, M-L -2.4, D-V 3+3.8 with respect to the interaural line for intrahippocampal injections. Injections were made i.p. at 30 mg/kg.

### Preparation of tissue extracts

Samples from whole brain, liver, or spleen or from cultured cells were homogenized on ice (with sonication for tissues) in T-PER reagent (Tissue Protein Extraction Reagent, Pierce Biotechnology, Rockford, IL). Protein concentration of these whole cell extracts was determined using the BioRad Protein Assay Reagent (BioRad, Hercules, CA) according to the manufacturer's instructions. Cytosolic and nuclear extracts of cortical neurons were prepared using the NE-PER kit (Pierce) according to the manufacturer's instructions. Both the T-PER reagent and the buffers in the NE-PER kit were supplemented with both a protease inhibitor cocktail (HALT, Pierce Biotechnology, Rockford, IL) and a phosphatase inhibitor cocktail (Phosphatase Inhibitor Cocktail 1, Sigma Life Science, St. Louis, MO).

### Western blots

Protein samples of either tissue extracts or individual cytosolic or nuclear extracts were loaded in equal amounts into individual wells of a 10% polyacrylamide-SDS gel and the proteins resolved using MOPS-SDS buffer. Proteins were transferred to a PVDF membrane (Millipore-Immobilon-P, Bedford, MA) for 2 h at room temperature, and then the nonspecific sites were blocked with 5% Dried milk in Tris-Buffered Saline containing 0.05% Tween-20 (TBST buffer) with gentle washing for 2 h at room temperature. The membranes were incubated overnight at 4°C in a fresh solution of 5% dried milk-TBST containing antiserum for p50 or p65 at optimized dilutions for each antibody. After the overnight incubation, membranes were washed three times with TBST (10 min each) and re-incubated with a goat anti-rabbit IgG-HRP secondary antiserum (Promega Corporation, Madison, WI) diluted in 5% dried milk-TBST for 4 h at room temperature. The membranes were washed three times with TBST (10 min each), reacted with the West Duro Chemiluminescent substrate (Pierce) and exposed to X-ray film to visualize protein present in the samples. Approximate molecular weights of the target proteins were determined by comparison to a BenchMark prestained protein ladder (Invitrogen).

### Immunocytochemistry (IC)

Neurons were fixed on coverslips in 4% PBS-paraformaldehyde for 30 min at room temperature, washed with PBS and then permeabilized with 0.1% Triton X-100 (Sigma) in PBS for 15 min at room temperature. The cells were blocked with PBS-0.1% Triton containing 5% Normal Goat Serum (NGS) for one hour at room temperature and incubated overnight at 4°C with the appropriate primary antiserum diluted in PBS-0.1% Triton-5% NGS. Antiserum tested was sc-372G (Santa Cruz) at 1:700 dilution. After washing with PBS-0.1% Triton, the cells were incubated for 2 h at room temperature with the anti-goat biotinylated secondary antibody followed by avidin, biotinylated enzyme, and diaminobenzidine using the Vectastain Elite ABC kit (Vector Labs, Burlingame, CA) for brown color reaction. Coverslips were inverted and fixed to slides with Permount for later examination under a microscope (Leica DMR) and CCD camera (CoolSnap cf, Photometrics, Tucson, AZ).

### Immunohistochemistry (IHC)

Mice were deeply anesthetized (pentobarbital plus chloral hydrate) and transcardially perfused with saline followed by freshly prepared phosphate buffered 4% paraformaldehyde. Brains were removed and stored in the same fixative followed by transfer to a 20% sucrose solution. Microtome-cut, 40-μm-thick, free-floating sections were processed as above. For IHC, prior to adding the primary antibody, control sections were incubated for 15 min in 3% hydrogen peroxide in PBS to quench endogenous peroxidases and then washed. Quenching had no effect on the staining outcome, so it was eliminated in remaining experiments. The step was omitted in the IC procedure because control experiments (incubating fixed cells in the Vectastain color reaction reagents) indicated that quenching did not affect the color reaction.

## Results

### Western blot tests of p65 antibodies in WT and KO tissue homogenates

**Figure 1:**
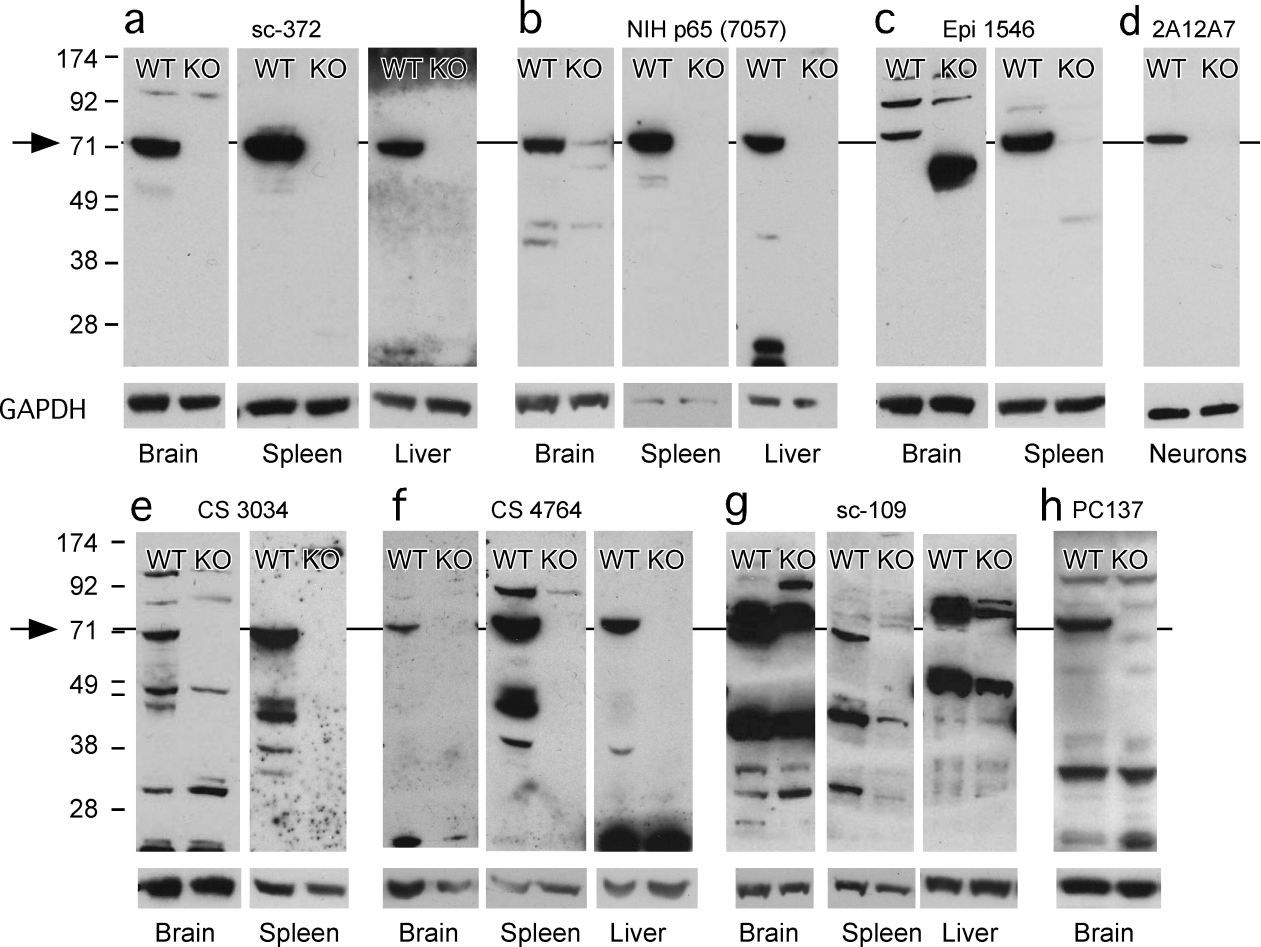
**Western blots show p65 antibodies that passed the test of specificity in p65 KO tissues**. Amounts of cell extract loaded per lane indicated after each antibody. a, sc-372R (brain 20 μg, spleen 20 μg, liver 20 μg); b, NIH p65 (brain 40 μg, spleen 20 μg, liver 20 μg); c, Epitomics 1546 (brain 20 μg, spleen 20 μg); d, Invitrogen 2A12A7 (neurons 10 μg); e, Cell Signaling 3034 (brain 20 μg, spleen 40 μg); f, Cell Signaling 4764 (brain 40 μg, spleen 40 μg, liver 40 μg); g, Santa Cruz sc-109 (brain 40 μg, spleen 40 μg, liver 40 μg); h, Calbiochem PC137 (brain 20 μg). The pattern of bands seen in h was identical to the patterns produced by the same antibody sold by Chemicon (AB 1604) and Rockland (100-4165) (not shown). Arrows point to locations in blots of the protein identified as p65. WT, wildtype; KO, knockout tissue.

**Figure 2:**
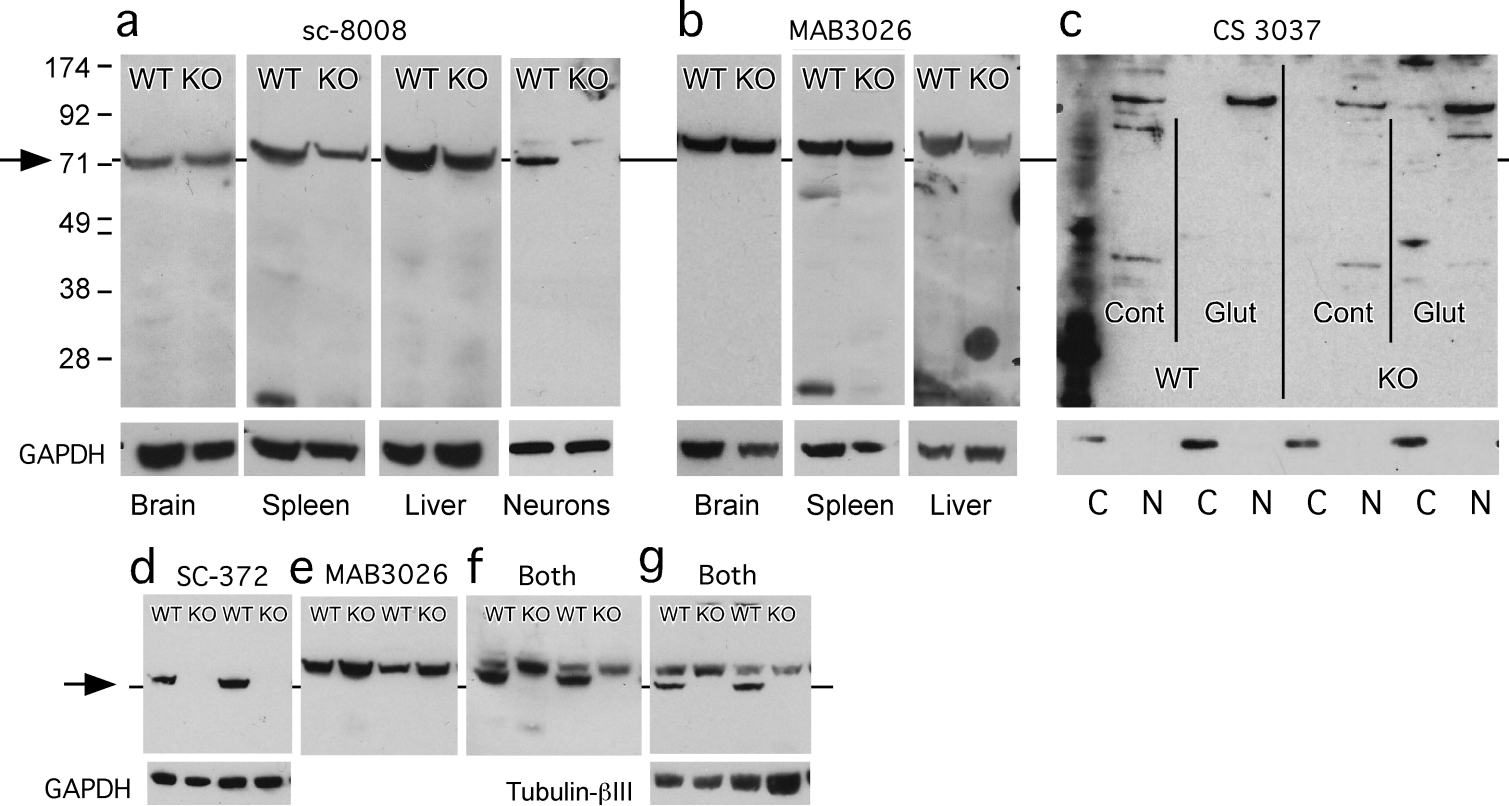
**Western blots show p65 antibodies that failed the test of specificity in p65 KO tissues**. a. The sc-8008 antibody showed staining at approximately the correct molecular weight, but it was present also in p65 KO tissues. Curiously, in protein from cultured neurons, the protein from KO cells was lacking. Protein load was 40 μg for brain, spleen and liver and 10 μg for neurons for both a and b. b. The MAB3026 monoclonal antibody against the p65 NLS showed a single band that was present in both WT and KO tissue. c. The CS 3037 antibody is raised against the phosphorylated (at Ser276) form of p65. The staining was almost entirely in the nuclear (N) fraction, absent in the cytosolic (C) fraction, and was induced in the high molecular weight range by glutamate (Glut) treatment (100 μM, 10 min) relative to unstimulated control (Cont). However, the bands were not present at the expected molecular weight for phospho-p65. Protein (5 μg per lane) is from cortical neurons. In d-g, proteins from cultured neurons were run to compare sc-372 and MAB3026. Compared with the band stained with sc-372 (d), the band stained with MAB3026 runs in the gel at a slightly higher molecular weight (e). The same gel was probed for MAB3026 (e), then stripped and re-probed for both antibodies simultaneously (f), or probed initially with both antibodies (g) to show that the single band marked by the MAB3026 antibody is not the p65 epitope.

There were several p65 antibodies that marked a single protein migrating in the gel at the correct molecular weight, and this marked band was absent in lanes containing protein from p65 KO tissue. These were Santa Cruz sc-372, NIH p65, and Invitrogen 2A12A7 monoclonal (Figure 1a, 1b, 1d). Another set of p65 antibodies marked proteins in multiple bands, including a band at 65 kDa that was absent in p65 KO tissue. These were Epitomics 1546, (Figure 1c), CS 3034 (Figure 1e), CS 4764 (Figure 1f), sc-109 (Figure 1g), Calbiochem PC137 (Figure 1h), and the NCI repository antibodies 1226, 1774, 1207, and 33 (not shown). Finally there were several antibodies that failed the KO test in that they marked proteins near the p65 molecular weight equally strongly in from WT and KO tissues. These were MAB3026, sc-8008, and CS 3037 (phospho-Ser276-p65) (Figure 2a-c). The data for sc-8008 are enigmatic because the antibody labeled a single band at the correct molecular weight only in WT protein when applied to neurons in culture but marked a similarly sized band from p65 KO protein when applied to tissues, suggesting that it recognizes a foreign protein that is not present in neurons. The blot for 3037 was done on nuclear and cytosolic extracts from cultured neurons that were unstimulated or glutamate stimulated (100 μM for 10 min). Glutamate was tested because of previous findings that glutamate is a stimulus for NF-κB activation in neurons [3].

The failure of the MAB3026 antibody was quite surprising, given that in early publications, then named α-p65M, it passed stringent tests for validity in cells transfected with a construct overexpressing p65 [10]. Therefore, we compared it closely with sc-372, which met the criteria for specificity in the p65 KO test. In one set of gels, neuronal proteins from p65 WT and KO were first probed with MAB3026, and then the same blot was stripped and re-probed with sc-372 (as well as GAPDH to assess the protein loaded). To a second set, the opposite was done; they were first probed with SC372 (Figure 2d), stripped and re-probed with MAB3026 (figure 2e) (as well as GAPDH to assess the protein load). The gel was then treated with both antibodies (Figure 2f). The results clearly show that MAB3026 marks a protein band that is different from the band marked by sc-372, both in its location and its presence in KO tissue. In a second test, a dual blot was made applying both MAB3026 and sc-372 primary antibodies at the same time, with tubulin-βIII as the load protein marker (Figure 2g); the same result was obtained. Both experiments clearly show differences in molecular weights of the proteins being detected by the two antibodies in the blots as well as the inability of MAB3026 to discriminate between WT and p65 KO tissue samples.

### Western blot tests of p50 antibodies in WT and KO tissue homogenates

**Figure 3:**
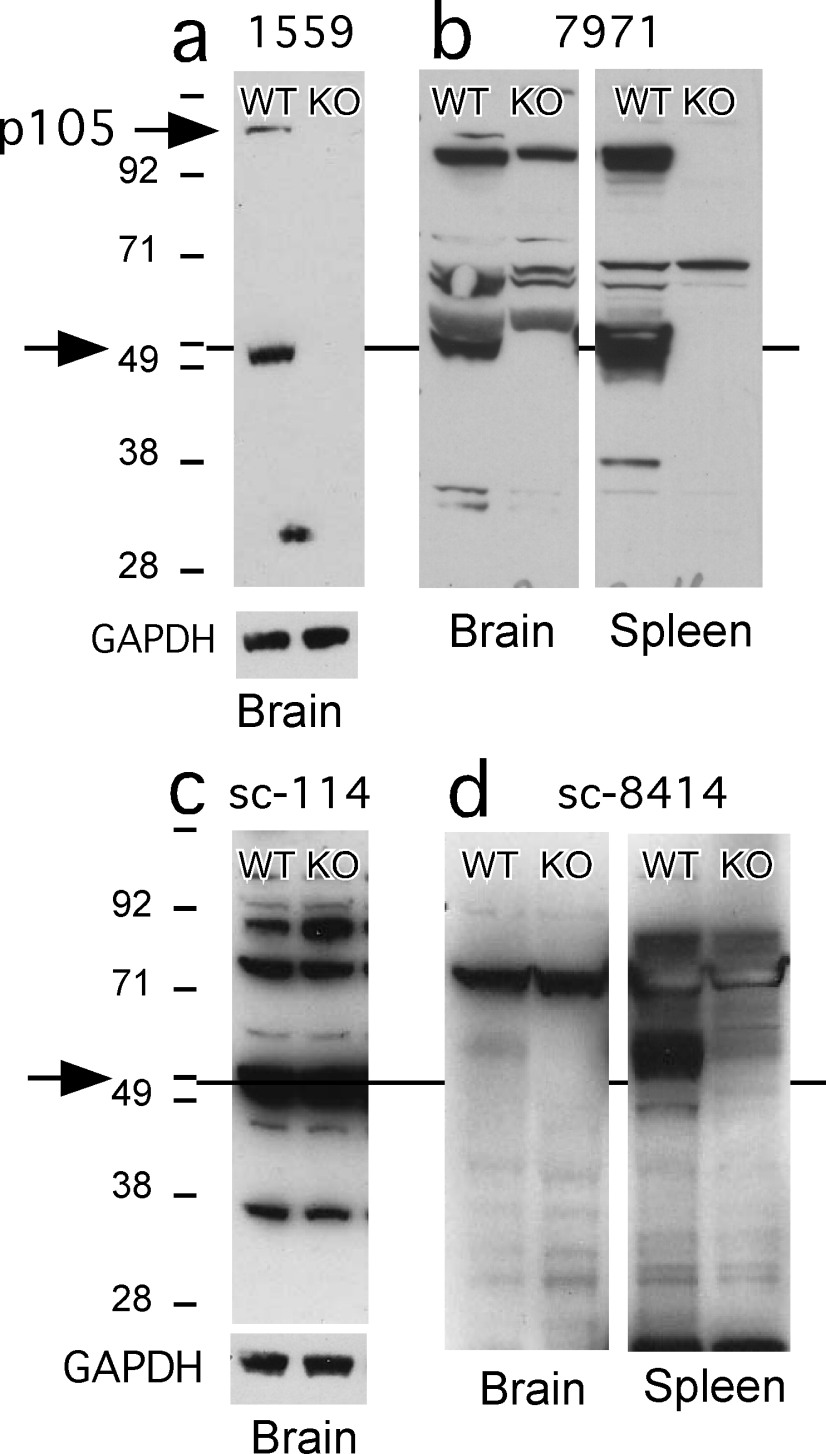
**Western blots show p50 antibodies that passed (upper) and failed (lower) the test of specificity in p50 KO tissues**. The protein load was 20 μg per well in a and 50 μg in b, c, and d. Arrows point to approximate 50 kDa MW or to 105 kDa as indicated in the upper blots.

The Epitomics 1559 rabbit monoclonal antibody marked a band at the correct molecular weight that was absent in p50 KO tissues (Figure 3a). As expected, it also marked a band at the molecular weight of p105, the precursor protein transcribed by the *nfkb1 *gene. Other p50 antibodies labeled multiple bands including a band at 50 kDa MW that was absent in p50 KO tissue. These included Abcam 7971 (Figure 3b) and the NCI repository antibodies 1157, 1263, and 1613 (not shown). Finally, p50 antibodies that failed the specificity test because they marked multiple bands and did not mark a band at 50 kDa that was absent in p50 KO tissue included sc-114 (Figure 3c), sc-8414 (Figure 3d), and Upstate 06-886 (not shown).

### Phospho-ser276-p65 antibodies

**Figure 4:**
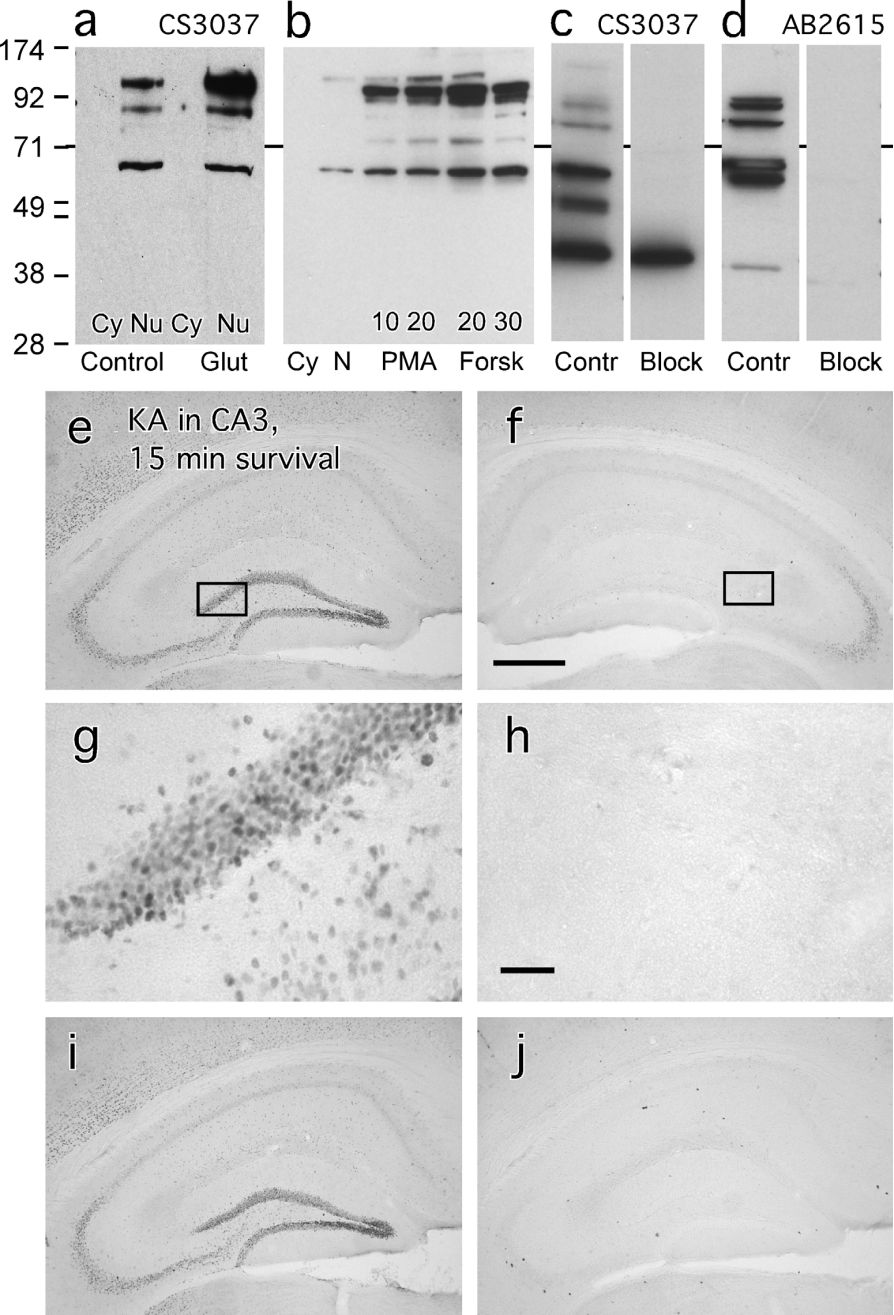
**Western blots and immunohistochemistry show nonspecific protein marking by antibodies raised against phospho-ser276-p65**. Antibody CS 3037 marking of proteins in gels run with extracts from hippocampal neurons in culture was exclusively nuclear, induced by 10 μM glutamate (a) or by 10 and 20 μM PMA or 20 or 30 μM forskolin (b) for 10 min. Marking of the nuclear proteins was blocked by the synthetic peptide used for immunization (2.5 μg per reaction in western blots) (c, d). However, the induced labeled proteins did not migrate at the molecular weight for p65. Immunohistochemical staining of the large molecular weight proteins (but not phospho-ser276-p65) marked by the phospho-ser276 antibody CS 3037 applied at 1:200 dilution is shown in e-g. Intrahippocampal KA injection caused a rapid (within 15 min) local neuronal response in granule cell and pyramidal neurons ipsilaterally (e, g) but not contralaterally (f, g). High-magnification views of the dentate gyrus on the left (g) and right (h) sides are taken from the boxed areas above. Preincubating the tissue with 1 μl/ml of a 1 mg/ml phosphorylated (j) or non-phosphorylated (i) p65 peptide fragment against which the antibody was generated demonstrated that only the phosphorylated peptide blocked the staining. Magnification bar in e = 200 μm for d,e,h, i. Bar in g = 20 μm for f, g.

There are two sites of p65 phosphorylation, on serine 276 and serine 536 [1]. The mitogen and stress-activated kinase-1 (MSK-1) and protein kinase A catalytic subunit (PKAc) pathways lead to ser276 phosphorylation resulting in further NF-κB activation [11-13]. The phospho-ser276 form of p65 is reported to reside exclusively in the cell nucleus [13]. In western blots, protein bands stained by the Cell Signaling 3037 antibody raised against phospho-ser276-p65 were confined to the nucleus, as expected (Figure 4a). However, the stained bands were not found at the expected position of p65 in the gel, rather, they appeared at a lower and several higher molecular weights. These upper bands were not protein complexes containing p65 because the proteins were thoroughly denatured before running on the gel. The material was also treated with phosphatase inhibitors, precluding degradation of the protein. A thorough proof of the failure of this and three other commercial phospho-ser276-p65 antibodies to recognize phosphorylated p65 in western blots of proteins from cell lines has been published [14]. Interestingly, stimulation of neuronal cultures with glutamate resulted in increased staining of the large molecular weight bands (Figure 4a). The bands could be induced also by treatment with phorbol 12-myristate 13-acetate (PMA) or forskolin (Figure 4b). Finally, the staining of multiple bands was prevented if the tissues were treated with the peptide against which the antibody was directed (Figure 4b). Similar results were obtained for Abcam's ab2615 antibody directed against a similar region of phospho-ser276-p65 (Figure 4c.). This antibody stained multiple bands, none of which were at the correct molecular weight for phosphorylated p65.

Similar results were obtained in IHC studies. Staining was induced by KA in hippocampus (Figure 4e); it appeared rapidly in neuronal nuclei (Figure 4g), and it was blocked by the phosphorylated peptide against which the antibody was raised (Figure 4j) but not by the unphosphorylated form of the same peptide (figure 4i) that was used to generate antibody 3034 sold by Cell Signaling. The blocking was completely effective in immunohistochemical studies of both tissues (Figure 4i and 4j) and cells (not shown).

### Use of the sc-372 p65 antibody for immunocytochemistry and immunohistochemistry

**Figure 5:**
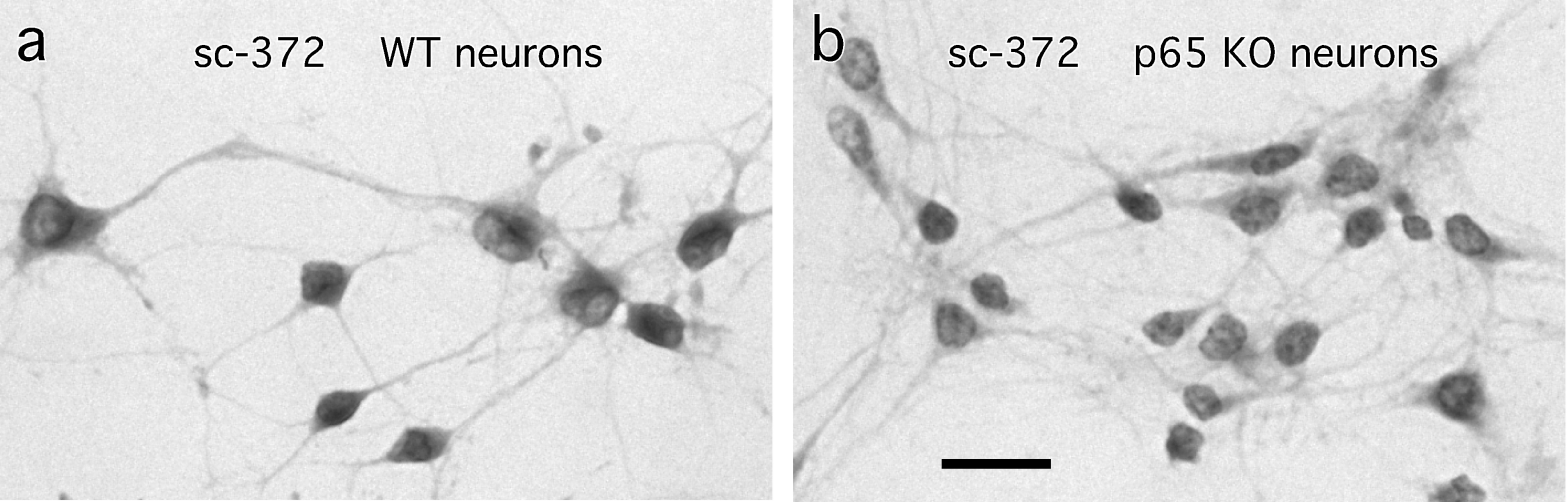
**Staining appearance is similar in cultured hippocampal neurons immunostained with the sc-372 antibody against p65**. a. neurons from WT mice. b. neurons from p65 KO mice. Primary antibody dilution was 1:700. Bar = 20 μm.

Primary hippocampal or cortical neurons grown in low density on coverslips were processed with the sc-372 p65 antibody for immunocytochemistry. In untreated cells from WT mice, brown reaction product filled the cell cytoplasm, including the larger processes (Figure 5a). The same pattern of staining was seen in cells from p65 KO mice (Figure 5b). In another set of experiments, cells grown on coverslips were processed for immunofluorescence. For all antibodies tested--sc-372, NIH p65, Epitomics 1546, CS 3034, and CS 4764--at a range of dilutions--1:200 to 1:20,000--staining was seen in the processes, cytoplasm, and nucleus of all cells, and no consistent differences were seen for staining patterns in either pure neurons (Figure 5) or mixed cells cultured from WT or p65 KO fetal brains (data not shown).

**Figure 6:**
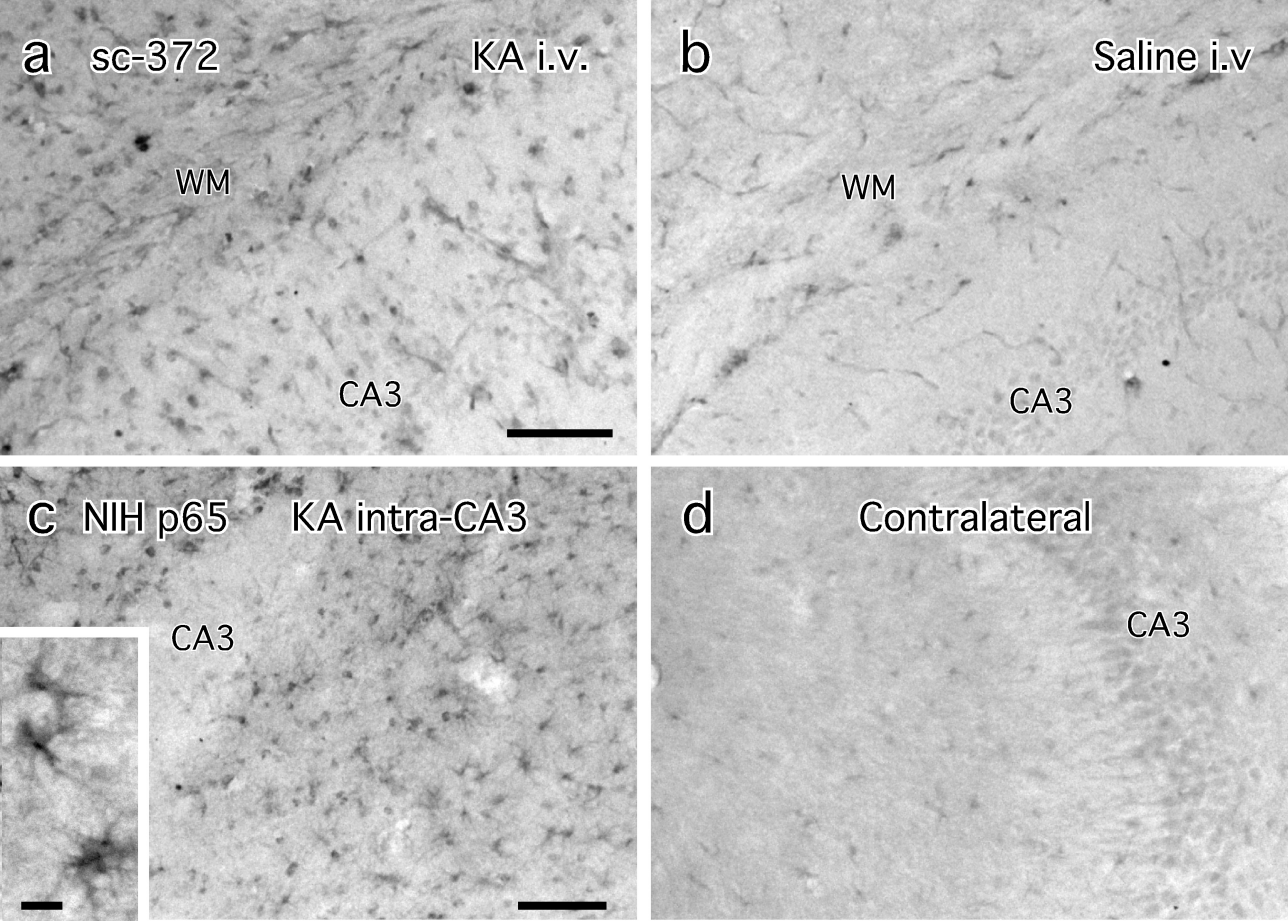
**Immunohistochemical staining with sc-372 and NIH p65 antibodies in hippocampus after kainic acid (KA) treatment**. In a and b, KA (a) or saline (b) was injected i.v. three days previously. In c-e, KA was injected into the left CA3 four days previously. The CA3 label is placed over the pyramidal cell layer in a-d. WM is the subcortical white matter. The inset shows darkly stained cells that appear to be astrocytes or microglia in c, based on morphology and size. Neurons are unstained or very lightly stained in all panels. Blood vessels are moderately stained in some sections, notably in a and b. Magnification bars in a and c = 100 μm for a,b and c,d. Bar in inset = 20 μm.

Microtome-cut free-floating sections from perfusion-fixed adult brain sections were also processed for immunohistochemistry (Figure 6). The two antibodies that showed high specificity in the western blot test, sc-372 and NIH p65, were applied to brain sections from animals that had been given KA to induce excitotoxicity in the hippocampus. Survival times in these experiments ranged from 15 min to 14 days. KA-induced effects were observed in tissues at the three- and four-day survival times, reminiscent of published data using the sc-372 antibody after similar kinds of treatments [15]. The altered staining consisted of moderate elevations in intensity in vascular endothelia and glial cells in the region of injury (Figure 6a, 6c) contrasted with either saline-injected control (Figure 6b) after i.v. injection or the side opposite the intrahippocampal KA injection (Figure 6d). The morphology of the glial cells most resembled astrocytes (inset, Figure 6c). Staining filled the cells, indicating both cytoplasmic and nuclear localization. Neurons showed no increased immunostaining.

## Discussion

### Cellular localization of p65 immunostaining in brain

Of the tested p65 antibodies, the polyclonal Santa Cruz sc-372 antibody showed the strongest, most repeatable pattern in western blots. In most blots, a single band appeared in the location marked by the 70-kDa lane marker. The single band was completely absent in the RelA knockout (p65 KO) tissue, in brain, liver, and spleen samples. A similar pattern was found with the NIH p65 antibody. The four antibodies that met this rigorous test of specificity were all raised against a portion of the C-terminus of p65, which includes its transactivation domain (TAD). In contrast, antibodies raised against the N-terminus or against the NLS region did not meet the criteria for specificity. Thus, it is apparent that the C-terminus contains antigenic peptide sequences that are unique to p65, whereas other regions of the molecule are not so selectively and strongly antigenic. The NLS region should be notably difficult to raise selective antibodies against because the aa sequences are conserved for a number of proteins that have NLSs. The N-terminal Rel homology domain (RHD) is conserved across other proteins, so this too may not be a suitable region for antibodies. Finally, not surprisingly, the rabbit monoclonal antibodies gave excellent results. They are new and have not been used in many publications to date.

The sc-372 antibody has been used in several publications to characterize NF-κB activity in the brain. A study by [15] examined the effects of intracerebroventricularly injected KA-induced NF-κB activity in the hippocampus. This study is one of few to show a western blot. The illustrated molecular weight range was confined to 51-80 kDa, and in that range was a single band. Intensity of the band increased in tissues from animals surviving from 1 to 7 days. Immunohistochemistry of the CA3 region of the hippocampus revealed induction of staining in glial cells with the appearance of astrocytes or microglia (co-labeling with both cell types was confirmed by double immunostaining with GFAP and CD11b, respectively), and the staining was found in both the nucleus and cytoplasm. This pattern of staining was closely mimicked by the staining with the NIH p65 antibody (Figure 6c-e). It also agrees with the astrocyte-like cellular staining in hippocampus 4 days after KA injury in another study using the sc-109 antibody [16], which we find to authentically mark p65 but also additional proteins in the western blot.

In human brain infarctions, a selective staining of glial cells was seen by Terai [17] using the sc-372 antibody. In the infarct area, microglial cells and blood vessels were also stained. The entire cytoplasm of the cell appeared stained, and such staining was largely absent outside the infarct area.

In a study of status epilepticus seizures, which are associated with neuronal loss, p65 staining with sc-372 was induced in astrocytes and blood vessels mainly in the hippocampus and entorhinal cortex [18]. Constitutive staining was minimal. There was no overlap of p65 staining and fluorojade staining of degenerating neurons.

Following immune challenge by i.p. IL-1β injection, Nadjar et al. used the sc-372 antibody for IHC in rats and mice to examine the cellular CNS response [19]. Background staining was low, and induction of staining was seen in non-neuronal cells of the choroid plexus, circumventricular organs, and blood vessels. Co-localization with astrocytes was positive. The temporal course of activation, peaking in the 1-2 hour range, was mirrored also by IκBα mRNA analyzed by RT-PCR. The localization, cell type, and timing of this response was rather perfectly matched by studies of IκBα mRNA induction visualized by ISHH after peripheral injections of lipopolysaccharide [20] or IL-1β [21]. IκBα is one of the family of inhibitory proteins that hold NF-κB in the cytoplasm until it is activated [1]. Activation results in degradation of IκBα, which is followed typically by increased transcription and synthesis of IκBα to replace the reserve of inhibitory molecules. Thus, IκBα mRNA induction is a reliable marker of NF-κB activity in cells [20]. Significantly, no published ISHH study has shown neuronal induction of IκBα mRNA in any model of brain injury or other form of CNS activation.

Other IHC publications are more difficult to interpret. Quite a few studies do not provide sufficient information in the Method section to allow determination of which specific antibody was used. A study by Gabriel [22] does not specify which Santa Cruz rabbit polyclonal p65 antibody was used (sc-109 or sc-372), but a western blot showed a single band, suggesting it was sc-372. Dilution of primary antibody was 1:1,000. In the study, control cortex showed staining of the cell cytoplasm in neurons, astrocytes and microglia. After an infarct, endothelium and glial cells were strongly stained in the ischemic area. Nuclear staining was predominant in astrocytes, microglia, and macrophages. In a different study of cortical injury using the sc-109 antibody for IHC, combinations of neuronal and non-neuronal cells were positively stained in the region of injury [23]. Similarly with this antibody, a diffuse neuronal staining pattern was replaced by a more specific staining of astrocytes and microglia in the region of a cortical injury induced by local injection of NMDA [24,25]. A study of ischemia in the gerbil showed exclusively glial (astrocytes and microglia) p65 staining in the hippocampus with the monoclonal antibody (sc-8008) from Santa Cruz [26]. This antibody showed specificity in cultured neurons in the KO test (figure 2a). Results must be interpreted with caution in work using p65 antibodies that detected a band at the correct molecular weight in WT but not KO tissues, but also marked bands at other mobilities; these other proteins might be stained in IHC material.

In summary, studies employing the antibody we have characterized here as the best one for localizing authentic p65 in brain have fairly consistently shown that p65 staining is not appreciably present in neurons and is not induced in neurons after various brain insults. Rather, p65 immunostaining is increased in endothelia, astrocytes, and microglia in these studies.

In contrast to the above summary, another group of studies shows predominantly neuronal p65 staining, both constitutively and after pathological insults. The most widely used antibody for IHC to depict this pattern of NF-κB activity in the brain is the MAB3026 antibody against "activated" p65. The original p65 antibody raised against the NLS region was a rabbit polyclonal made by P. A. Baeuerle [27] and named α-p65NLS. Later, an affinity purified monoclonal antibody of the IgG3 isotype, called α-p65M [28] and α-p65Mab [29], was used by the Kaltschmidts in early publications and then distributed (as clone 12H11) by Boehringer-Mannheim (1697 838), Roche (1697838), and finally licensed by Chemicon (Millipore) and sold as MAB3026. It was made against the human p65 sequence CDTDDRHRIEEKRKRT (NLS underlined). The authenticity of the monoclonal antibody was determined by western blot [10]. Interestingly, in normal 293 cells, a single band appeared at around 67 kDa, but in 293 cells with elevated p65 levels due to transfection with a CMV-p65 plasmid construct, a dense band appeared at 67 kDa and a lighter band appeared just below it, at around 65 kDa. Similar multiple banding patterns were seen in western blots in a subsequent publication [30]. In light of the present western blot data showing that the band marked by MAB3026 runs just above the band marked by sc-372, it is possible that the faint lower band is the authentic p65, and the major upper band is an unknown protein with biological properties very similar to p65.

In the early years, the MAB3026 antibody was used to support the argument that there was constitutive and induced NF-κB activity in neurons [28,31]. Very high concentrations of the antibody were typically used--in the 1:30 to 1:150 dilution range. With this antibody, injury-induced neuronal staining was shown in cerebral cortex and the hippocampal CA fields at 1-3 days after middle cerebral artery occlusion [32] or 1-2 days after traumatic brain injury (TBI) [33] and in the hippocampal CA fields 1 day after preconditioning by transient ischemia or KA administration [34]. In human brain, the MAB3026 antibody strongly stained cortical neurons in the ischemic area following a stroke [35]. These data are very different than the published data in similar models described above that used the sc-372, sc-109, and sc-8008 antibodies.

Following peripheral lipopolysaccharide (LPS) administration eliciting an inflammatory response in the brain, ISHH showed strongly induced IκBα mRNA expression in non-neuronal cells of the meninges, blood vessels, brain parenchyma, and circumventricular organs [20,21]. In striking contrast, in this same LPS administration paradigm, IHC of p65 staining using MAB3026 showed exclusively neuronal induction in one brain region adjacent to one circumventricular organ [36]. It is not known what factors would produce this pattern of staining.

### Cellular localization of p50 immunostaining in brain

Of the tested p50 antibodies, the Epitomics 1559 rabbit monoclonal antibody raised against a peptide sequence located between aas 340 and 370 of p50 was the best antibody in the study. It is new and has not been used in published studies of brain localization or activity. In contrast, the Santa Cruz sc-114 antibody, raised against the NLS region at aas 357-365 to generate an "activated p50" antibody, was clearly not directed against p50 in our gels. This fact has already been published [37]. In that study, thymic whole cell extracts from WT and p50 KO mice were used to show that the sc-114 antibody produced several nonspecific bands of incorrect molecular weight that appeared identically in both WT and p50 KO tissues run on western blots. Despite the early knowledge that sc-114 did not recognize p50, several publications have used it to show neuronal p50 localization in hippocampal CA cells following KA- or NMDA-induced damage [38-41]. These results are counter to the outcomes, described above, using the sc-372 antibody against p65.

### The cautionary story of antibodies to phospho-ser276-p65

A study has already been published showing the lack of specificity of this and other phospho-ser276-p65 antibodies in a number of cell lines [14]. Our findings extend the list of targets, namely primary neurons and brain cells, against which the 3037 antibody does not recognize Ser276-phosphorylated p65. Staining was completely blocked by the phosphorylated peptide against which the antibody was raised. Peptide blocking is a standard test for peptide specificity, but it only shows that the antibody is specific for the peptide it was raised against and not the native protein containing the peptide sequence. The case of a single band migrating on a western blot at the appropriate molecular size being effectively competed away by the blocking peptide can be viewed as an adequate test of the specificity of the antibody. However, when multiple bands of inappropriate molecular size are present on the western blot and they are all competed away by the blocking peptide, a question of the specificity of this antibody is raised as it appears that the antibody is recognizing nonspecific epitopes in unrelated proteins. Thus, while passing this traditional test of specificity, 3037 failed to mark a protein in the gels at the correct molecular weight (at around 65-70 kDa in our gels) that was also absent in the p65 KO mouse.

The amino acids surrounding ser276 of p65 contain sequences that are also found in proteins that are phosphorylated by a number of enzymes, so it is possible that the antibody marked unknown phosphorylated proteins of large molecular weight. Indeed, these proteins were very responsive to glutamate treatment as indicated in western blots by intensified bands of nuclear but not cytoplasmic proteins appearing within minutes of glutamate stimulation, in immunohistochemically stained hippocampal neurons following in vivo KA treatments (Figure 4), and in nuclei of hippocampal neurons in culture after glutamate treatment (not shown). Glutamate acts through Ca^++ ^and enzymes like PKC and PKA to phosphorylate nuclear proteins. In one study, glutamate stimulated the production of PKC-phosphorylated proteins at 48, 87, and 120 KDa molecular weight in hippocampal neurons [42]. We found that the bands at around 90 and 130 kDa were dose-dependently increased in intensity by treatment with PMA (which activates PKC) and with forskolin (which activates PKA). Further work is needed to identify the actual targets of the 3037 antibody.

### Neuronal immunostaining with p65 and p50 antibodies

We show here that even antibodies that pass the test of specificity on western blots still label cells nonspecifically when used for IHC. The probable reason for this is that the levels of p50 and p65 in neurons are so low that they cannot be detected by this method. Raising the concentration of the primary antibody to the point that cellular staining becomes visible amounts to nonspecific staining, which we see even at 1:500 dilution of authenticated antibodies. Antibodies that mark additional proteins in the western blots are even more difficult to interpret when used for IHC, especially in reports of changes in staining levels induced by various manipulations. It is unlikely that the changes measured are due to alterations in levels of p65 or p50. Any of the other proteins recognized by these antibodies might contribute to the immunohistochemical outcome. We (Listwak et al., unpublished data) and others [43] have found that NF-κB in neurons is rather unresponsive to stimulatory challenges of all kinds. Consequently, recent reports of altered NF-κB activity inferred from changes in subunit immunostaining following mild challenges like electrical stimulation of afferent pathways [44], passive avoidance learning [45], or psychological stress [46] need to be viewed with great skepticism. These finding are further rendered suspect by the use of the MAB3026 antibody [44,45,47], the use of unidentified Santa Cruz p65, p50, and IκBα antibodies without designation of catalog numbers [46], and by the fact that the brain focus of the studies is the hippocampus, which has layers of very tightly packed neurons that render the appearance of specific staining, especially at low magnification, because of the high neuronal packing density. With antibodies such as sc-372 and NIH p65, we find that neurons are the least stained and most unresponsive of all the cellular phenotypes in the hippocampus but are visible because of the high packing density (note CA3 cell layer in Figure 6b, 6d).

The close functional association of p50 with p65 should result in similar immunohistochemical staining patterns in tissues following manipulations. A few studies have used p50 antibodies other than sc-114 for IHC in brain. Some used antibodies distributed by Warner Greene (Gladstone Institute). A p50 antibody named Ab 392 raised against the N-terminus was used in several IHC publications following its characterization in COS cells [48]. A western blot of brain extracts showed a band at 50 kDa plus multiple additional bands [49]. Dying hippocampal neurons were immunostained in that study and in a similar study that also used an N-terminus-directed p65 antibody from Greene called Ab 567 [50]. In another study by the same group, after cerebral infarct, both p50 and p65 antibodies from Greene stained neurons of all types in the infarct area with little staining outside the infarct area [51]. Neuronal staining with the p50 Ab 392 antibody appeared in one study in which every neuron of the medial septum was immunostained in the normal young brain, and the staining did not change following deafferentation by fornix transection [52]. It is difficult to assess the significance of these studies without knowing whether or not the antibodies were really specific for p50. Further work with authenticated p50 monoclonal antibodies is warranted.

A p50 antibody made by Mariagrazia Grilli (CNR Institute of Neuroscience, Milan, Italy) but not commercially available passed the test of marking single bands at 50 and 105 kDa in a western blot that were absent in p50 KO tissue, and it was used to show staining of radial glial cells in the subventricular zone and in the rostral migratory stream [53]. The authors did not show absence of such immunostaining in p50 KO tissue, however.

## Summary and conclusions

Widely used antibodies raised against the NF-κB subunits p65 and p50 were subjected to the test of specificity in WT and KO tissues, and several of the antibodies that failed the test of specificity are the ones that have been used in published studies to argue for neuronal activation of NF-κB in the brain following neurotoxic or other challenges. Antibodies that rigorously passed the specificity test show little specific neuronal immunostaining either in cell culture or brain, whereas they do mark non-neuronal cells in brains subjected to toxic or excitatory challenges. The data help to explain confusion in the literature about the role of NF-κB in neuronal function and injury. The lack of neuronal NF-κB response observed with the authentic p65 antibodies is a finding consistent with studies employing in situ hybridization histochemistry showing non-neuronal localization of induced IκBα mRNA expression in injury, infection, and pain models [20,54-56] and of known NF-κB-regulated genes in similar models [57-61]. Future studies with validated monoclonal antibodies and molecular tools such as a knock-in p65-GFP fusion protein [62] (but note the caveats associated with this approach [63]) will provide the proper means to address the cellular localization of NF-κB activation in the brain.

## Competing interests

The authors declare that they have no competing interests.

## Authors' contributions

MH conceived of the project, advised on experiments, prepared the figures, and wrote the manuscript. PR performed in vitro experiments, western blots, and immunohistochemistry of neuronal culture. PB performed animal surgeries and immunohistochemistry of brain sections. SL performed western blots and immunofluorescence. All authors read and approved the final manuscript.
